# Osteopathic Manipulative Treatment in Dysmenorrhea: A Systematic Review

**DOI:** 10.7759/cureus.52794

**Published:** 2024-01-23

**Authors:** Paige E Bonner, Heather A Paul, Rohit S Mehra

**Affiliations:** 1 Osteopathic Medicine, Nova Southeastern University Dr. Kiran C. Patel College of Osteopathic Medicine, Clearwater, USA; 2 Osteopathic Medicine, Nova Southeastern University Dr. Kiran C. Patel College of Osteopathic Medicine, Fort Lauderdale, USA

**Keywords:** osteopathic manipulative treatment, endometriosis, polycystic ovarian syndrome, dysmenorrhea, viscerosomatics, osteopathic manipulative medicine, spinal facilitation

## Abstract

The majority of women experience dysmenorrhea during their lifetime. The current standard-of-care treatment consists of nonsteroidal anti-inflammatory drugs, oral contraceptive pills, or intrauterine devices. Osteopathic manipulative treatment (OMT) is a beneficial tool for improving non-musculoskeletal (non-MSK) conditions such as migraines, gastroesophageal reflux disease (GERD), and anxiety. OMT should be utilized to improve other non-MSK conditions, such as dysmenorrhea. The current review aims to evaluate the effects of OMT in women with dysmenorrhea. An extensive search was conducted in Cumulative Index to Nursing and Allied Health Literature (CINAHL), MEDLINE, Biomedical Reference Collection: Comprehensive, and Nursing & Allied Health Collection: Comprehensive from inception to June 2022. Studies evaluating the use of OMT in patients with dysmenorrhea were included, while editorial/opinion articles were excluded. Three independent reviewers evaluated the studies. Ten studies evaluating the use of OMT in patients with dysmenorrhea were included. Overall, OMT was shown to provide relief of symptoms, including back and menstrual pain; however, there was no guideline on which OMT techniques are the most successful. Numerous positive effects were found, including a reduction in the duration of pain, reduction of pain intensity, and reduction of analgesic use. However, the low number of studies supports the need for further investigations. Dysmenorrhea patients could benefit from a prospective randomized controlled trial targeting spinal facilitation and viscerosomatic reflexes to decrease pain duration, pain intensity, and analgesic use. Non-MSK-focused OMT has a large body of mostly anecdotal evidence for relief of conditions such as migraine, GERD, and anxiety. It has helped when traditional standards of care have failed. Non-MSK-focused OMT research represents a relatively untouched field of research that can have a profound and positive global impact, particularly in areas with poor income/healthcare access.

## Introduction and background

An estimated 45-93% of reproductive-aged women around the world experience dysmenorrhea, including abdominal cramping, back pain, diarrhea, and vomiting [1]. Primary dysmenorrhea is defined as pelvic pain that occurs without known disease, while secondary dysmenorrhea is due to an underlying disease such as endometriosis or polycystic ovarian syndrome [1,2]. The etiology is attributed to an increase in prostaglandins during the menstrual cycle [3]. Pain and discomfort are typically highest on the first and second days of menstruation [1]. The pain and symptoms reduce women’s quality of life and their ability to complete activities of daily living [2]. Today, the first-line treatment for primary dysmenorrhea is nonsteroidal anti-inflammatory drugs (NSAIDs), of which 18% of patients are unresponsive to [1,3]. NSAIDs are known to have adverse effects such as gastrointestinal toxicities, cardiovascular risk, renal injury, and hepatotoxicity. The second-line treatment is hormonal therapy with oral contraceptive pills (OCPs) or intrauterine devices (IUDs). Combined OCPs have demonstrated effectiveness in reducing dysmenorrhea severity [4]. According to Choksey et al., after 12 months of OCP use in the treatment of dysmenorrhea, 12% of patients continued to experience pain [5]. Adverse reactions to estrogen contraceptives include nausea, vomiting, headaches, breast tenderness, and changes in body weight. Additionally, with progesterone OCPs, patients can experience acne, weight gain, increased hair growth, and depression [6]. 

An additional treatment is osteopathic manipulative medicine (OMM). OMM has been in use since the founder, Andrew Taylor Still, opened his first osteopathic medical school in 1892 [7]. The osteopathic approach to healing involves a holistic view of healing and caring for patients, utilizing manipulations such as high-velocity low-amplitude (HVLA) thrusts, lymphatic drainage techniques, myofascial release (MFR), muscle energy (ME), and counterstrain (CS). Osteopathic manipulative treatment (OMT) is a non-pharmacological intervention utilized to improve joint function, muscle tension, and viscerosomatic reflexes [8]. Viscerosomatic reflexes are TART (Tenderness, Asymmetry, altered Range of motion or tissue Texture changes) changes found at specific regions of the body caused by potential visceral pathologies [9]. OMT involves palpation of these TART changes and applies the manipulations to relieve symptoms and treat structural, lymphatic, or autonomic dysfunctions [10]. Dysmenorrhea patients often have viscerosomatic reflexes in the T10-L2 region, which can be effectively treated with OMT. There are minimal side effects; the most common side effect is pain immediately following treatment, occurring in 2.5% of patients [11]. This review aims to explore the use of OMT in the treatment of dysmenorrhea because of the side effects caused by the current pharmacological treatments. 

OMT is also particularly beneficial in areas with poor income/healthcare access because physicians can teach patients and families how to correctly perform some techniques at home. According to Rapp et al., 2.12% of women reported they were unable to access healthcare and 8.14% had no health insurance [12]. OMT can be especially helpful when patients do not have easy access to medications in rural or low-income communities.

## Review

Materials and methods

The following electronic databases were searched from their inception to June 2022: Cumulative Index to Nursing & Allied Health Literature (CINAHL) Complete, MEDLINE, Biomedical Reference Collection: Comprehensive, and Nursing & Allied Health Collection: Comprehensive. The search strategy was conducted using different combinations of the following terms: dysmenorr*, osteopathic manual therapy, osteopathic manip*, omt, cranial sacral treat*, and craniosacral. This systematic review includes randomized controlled trials (RCTs), controlled before-after (CBA) studies, case reports, and systematic reviews. In contrast, excluded studies were editorial/opinion contributions and one abstract that reported the same information included in one of the RCTs. 

Studies involving any type of OMT techniques were included. Techniques included HVLA, MFR, ME, CS, sacral inhibition, lymphatic pump, soft tissue (ST), and balanced ligamentous tension (BLT). No studies were included that used other types of manual medicine. The control groups varied per study, including controls that received no osteopathic treatment and controls that received sham treatment; however, no studies were excluded based on the presence/absence/type of control group. 

The patient population inclusion criteria were female patients of any age with primary or secondary dysmenorrhea. The primary outcome of the current systematic review was to evaluate the effectiveness of OMT in women with dysmenorrhea compared to any control group. All articles were screened based on abstract and titles. The data screening and selection process was carried out by three independent reviewers. The Joanna Briggs Institute critical appraisal tools (JBI, 2015) were utilized to assess the risk of bias, which classified articles as high risk=score less than 50%, moderate risk=50-70%, and low risk=scores greater than 70%. Only articles with a low risk of bias were included.

Results

**Figure 1:**
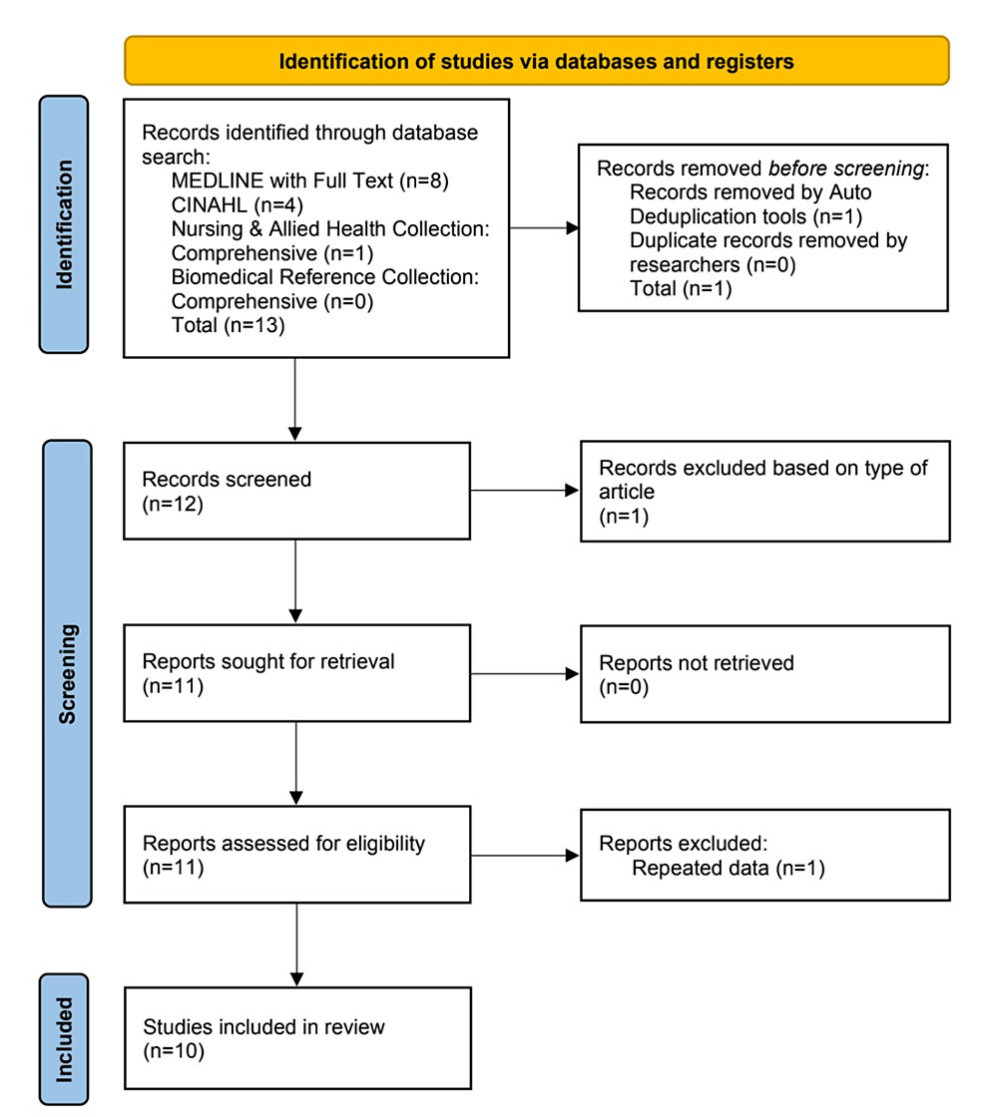
PRISMA chart of the study selection process CINAHL: Cumulative Index to Nursing and Allied Health Literature; PRISMA: Preferred Reporting Items for Systematic Reviews and Meta-Analyses

Electronic search from inception to June 2022 resulted in 13 articles identified. Twelve articles were screened, and two did not meet the inclusion criteria; therefore, 10 articles were used in this systematic review (Figure 1). 

Two articles were excluded [13,14]. One was an editorial/opinion piece, and the other was an abstract that reported the same data that was included in one of the RCTs. Four articles were RCTs, three were case reports, one was a systematic review, one was an evidence-based review, and one was a controlled before-after (CBA) study. Several different osteopathic techniques were used in each of the 10 studies evaluated (Table 1). 

**Table 1:** Types of osteopathic manipulation techniques used and their associated results HVLA: high-velocity low-amplitude; EMG: electromyography; OMT: osteopathic manipulative treatment; MFR: myofascial release; CS: counterstrain; ST: soft tissue; ME: muscle energy; VAS: Visual Analog Scale; PPT: pressure pain threshold; BLT: balanced ligamentous tension; WaLIDD: Working ability, Location, Intensity, Days of Pain, Dysmenorrhea; API: Average Pain Intensity; DDP: Days of Dysmenorrheal Pain

Study	Osteopathic techniques	Results
Boesler et al. [15]	HVLA	EMG in lumbar extension showed significant decrease in activity with OMT. Lab testing found no difference in total creatinine, total lactate dehydrogenase, or myoglobin/creatinine ratio.
Emo and Blumer [16]	MFR	Decrease in the duration of pain during a menstrual cycle when compared to the control.
Hitchcock [8]	Sacral inhibition, structural pressure, lymphatic pump	Decrease in menstrual disability, menstrual pain, and associated back pain.
Matsushita et al. [9]	MFR, CS, ST, ME, suboccipital release, abdominal plexus release, joint articulation	Adult onset of secondary dysmenorrhea improvement from stages 2-3 to stages 0-1 over six months after undergoing six OMT sessions.
Molins-Cubero et al. [17]	HVLA	Decrease in VAS and increase in PPT on the left sacroiliac joint. Serotonin, adrenaline, and dopamine levels increased and noradrenaline decreased after intervention.
Origo et al. [18]	Unwinding techniques, lumbosacral decompression, pelvic floor release, HVLA, BLT	Pain reduction of 50% during the menstruation week, WaLIDD improvement of 70%, and complete recovery from dyspareunia.
Ruffini et al. [10]	HVLA, MFR, ST, ME, rib raising, CS, lymphatic pump	Decrease in pain grade and analgesic use.
Schwerla et al. [19]	Appropriate techniques based on the dysfunction identified	API decreased from 4.6 to 1.9 in the intervention group vs 4.3 to 4.2 in the control group (p<0.005). DDP also decreased from 2.2 to 0.2 days in the intervention group vs 2.3 to 1.9 in the control group (p=0.002). A positive impact on quality of life was observed only in the intervention group.
Vlachos and Lagattuta [20]	HVLA, ME, MFR, BLT	Reduction in menstrual pain and duration.
Yosri et al. [21]	Visceral manipulation	Statistically significant improvement (p=0.024) in the severity of menstruation-related problems.

Description of Control

Out of the 10 studies, only 50% incorporated a control group. Different controls were used in each of these studies. In the CBA study, two-thirds of the participants received treatment during their menstrual cycle, and then during a different menstrual cycle, they were placed in a non-treatment group [15]. Two of the RCTs used a control group that did not receive any osteopathic or visceral manipulation [19,21]. One of the RCTs used a control group that received sham manipulation [17]. The last RCT used a control group that did not receive OMT, but did receive pharmacotherapy of ibuprofen or naproxen only [16].

Study Outcomes

The treatments each utilized different OMT techniques to treat women as shown in Table 1 and different techniques to evaluate the alleviation of symptoms such as the Visual Analog Scale (VAS), analgesic use, Polycystic Ovarian Syndrome Quality of Life (PCOSQ) Scale, menstruation distress, Average Pain Intensity (API), Days of Dysmenorrheal Pain (DDP), and Brief Pain Inventory (BPI) Questionnaire. Two studies examined a single technique in the treatment of dysmenorrhea [16,17]. The RCT that used HVLA at the sacroiliac joint found a decrease in VAS and an increase in the pressure pain threshold (PPT) on the left sacroiliac joint, and serotonin, adrenaline, and dopamine levels increased and noradrenaline decreased after intervention [17]. The other RCT demonstrated MFR improved pain intensity equally when compared to a control group that was administered NSAIDs; however, MFR decreased the duration of pain during a menstrual cycle in the treatment group [16].

The last two RCTs completed a holistic approach and treated each patient with a combination of treatment modalities. One RCT with 53 patients showed a decrease in API from 4.6 to 1.9 in the intervention group vs 4.3 to 4.2 in the control group (p<0.005). DDP also decreased from 2.2 to 0.2 days in the intervention group vs 2.3 to 1.9 in the control group (p=0.002). A positive impact on quality of life was observed only in the intervention group [18]. The final RCT examined females with polycystic ovarian syndrome and utilized OMT in conjunction with calorie-restricted diets vs only calorie-restricted diets [21]. The PCOSQ scoring evaluated other symptoms besides dysmenorrhea and therefore did not indicate any analysis of the dysmenorrhea symptom alleviation alone.

The CBA study used HVLA and tested the erector spinae muscles with electromyography (EMG), in addition to lab testing of creatinine, lactate dehydrogenase, and myoglobin/creatinine ratio to evaluate symptomatic relief [13]. When examining EMG in the lumbar extensors, there was a significant decrease in activity post OMT; however, there were no significant differences in the lab values. The study was limited to 12 participants and evaluated OMT in a single cycle of menstruation. To establish a control group, eight of the original participants repeated the protocol in a later menstrual cycle replacing OMT with rest. 

One case report examined a patient with dysmenorrhea secondary to trauma to the pelvis. After the four OMTs every two weeks, there was a pain reduction of 50% during the menstruation week, related disability (WaLIDD) improvement of 70%, and complete recovery from dyspareunia [18]. The second case report observed a reduction in the classification of the patients’ secondary dysmenorrhea from stages 2-3 to 0-1 after six OMTs [9]. The last case report treated a patient who had different leg lengths. The patient received OMT and a leg lift to correct the alignment, which showed progressive improvement in the patient’s symptoms of dysmenorrhea [8].

The evidence-based review utilized data from two RCTs to answer the question: Is OMT effective in reducing primary dysmenorrhea [20]? The 2017 Italian RCT included an intervention group of OMT every five days for two menstrual cycles and a control group of light touch therapy, also known as sham OMT. The OMT group had significantly lower pain levels, lower mental component scores, and a decrease in pain intensity [20]. The 2014 German RCT included an intervention group of OMT using HVLA, ME, MFR, and BLT and a control group without OMT. The OMT group had a significant reduction in menstrual pain and reduction in duration, but no significant difference was noted in quality of life [20].

This systematic review examined women after menarche in all gynecologic and obstetric conditions including pregnancy, labor, infertility, dysmenorrhea, pelvic pain, and menopause [10]. It concluded that OMT can be considered effective on pregnancy-related back pain, but effectiveness is uncertain in all other gynecologic and obstetric conditions, such as dysmenorrhea [10]. It did report that a decrease in pain grade and analgesic use was observed in subjects with dysmenorrhea who received OMT; however, due to small sample sizes, low number of studies published, and high risk of bias, results should be further assessed [10].

Discussion

This systematic review of 10 articles includes 299 patients and shows OMT is efficacious in treating dysmenorrhea symptoms, including abdominal cramping, back pain, diarrhea, and vomiting. It also demonstrates a reduction in pain duration, pain intensity, and analgesic use. OMT is an additional treatment to the current standard-of-care options and provides symptomatic relief as well as improves quality of life. The current standard of care is pharmacological treatment (NSAIDs, OCPs, and IUDs), which is associated with numerous side effects, such as nausea, vomiting, weight gain, gastrointestinal toxicity, renal toxicity, hepatotoxicity, and cardiovascular risk. OMT is associated with minimal side effects, the most frequent being pain immediately following treatment, occurring in only 2.5% of patients [11].

Osteopathic vs Pharmacologic Interventions

Among the 10 studies evaluated, only one study utilized pharmacologic treatment as the control [16]. Group A received OMT and Group B received ibuprofen or naproxen. Both the control and osteopathic treatments demonstrated statistically significant improvement (p<0.05) in pain intensity and pain duration [16]. By comparing the osteopathic treatment to NSAIDs, it is discernable that both treatments are effective in relieving dysmenorrhea symptoms. Using OMT could be advantageous to treat dysmenorrhea as it would avoid the known adverse effects of NSAIDs such as gastrointestinal toxicities, cardiovascular risk, renal injury, and hepatotoxicity. The cyclic nature in which dysmenorrhea symptoms occur may require long-term NSAID use. OMT as an effective therapy could decrease the overall quantity of NSAID use in reproductive-aged women. Additionally, 18% of NSAID users failed the treatment, further demonstrating OMT should be recommended [3]. 

The other nine articles evaluated have no comparison to the current standard of care. The case reports show isolated examples that highlight beneficial outcomes when treating dysmenorrhea with OMT [8,9,18]. Both the CBA study and the remaining RCTs provide additional data obtained from larger sample sizes [13,17,19,21]. Although these studies did not provide a comparison to NSAIDs, their value lies in showing reproducibility and decreasing the margin of error. 

Limitations

Each article highlighted the efficacy of OMT in the treatment of dysmenorrhea. However, each study utilized different qualitative and quantitative analyses to report changes in patients’ symptoms. While this encompasses a robust list, it makes comparing the study results more difficult. Sixty percent of the articles gave questionnaires to evaluate treatment effects. This subjective measure means that pain modulators such as stress and emotion can influence the patients’ evaluation of their pain. All of the studies that used questionnaires showed OMT improved dysmenorrhea symptoms. Only two of the studies used the VAS to measure pain intensity [16,17]. While these two studies can be compared to each other, future investigations with consistent quantification tools would be beneficial so that a greater number of patients can be compared.

The most important factor to call attention to is the limited number of patients examined in each study, along with the small number of articles available in this field of study. The largest RCT evaluated 60 patients, and the smallest RCT assessed only 30 patients [16,21]. This limits the ability to apply the findings to the larger population of women experiencing dysmenorrhea. 

In addition, only one of the studies compared OMT to the current standard of care using NSAIDs. To determine the clinical significance of OMT, more research would be needed to evaluate its effectiveness and encourage providers to recommend OMT. If additional studies are performed, a guideline of recommendations could be created that would help instruct providers on how best to alleviate these symptoms in their patients with dysmenorrhea. The last concern regarding this review is that there are two types of dysmenorrhea and depending on the cause of symptoms, there may be differences in the efficacy of OMT. Of the 10 studies, three studies examined secondary dysmenorrhea [9,18,21]. Separating and comparing the two types of dysmenorrhea and the efficacy of OMT would enhance the ability to understand which patient populations would benefit and to what degree. 

Future Considerations

It is well-known in osteopathic medicine that many times when a patient presents with pain, it can be a result of viscerosomatic reflexes. Afferent pathways originating from the viscera are transmitted through the dorsal horn into the spinal cord [22]. The synapses lead to efferent nerves acting on the skeletal muscles and blood vessels causing the viscerosomatic reflexes [22]. Dysmenorrhea patients can present with viscerosomatic reflexes from the uterus and endometrium in the following ranges: sympathetic: T10-L2 and parasympathetic: pelvic splanchnic S2-S4 [15]. These patients can also experience gastrointestinal tension which corresponds to viscerosomatic reflexes in these regions: sympathetic: T5-T9 and T10-L2 and parasympathetic: vagus (OA, AA, C2) and pelvic splanchnic S2-S4 [8,15]. Targeting OMT towards those regions has the potential to impact these patients' lives by decreasing their pain duration, pain intensity, and gastrointestinal dysfunction. Determining a specific regimen of OMT would help guide physicians when caring for dysmenorrhea patients. 

When conducting future investigations, spinal facilitation should also be explored. Spinal facilitation is a concept that describes how a specific region of the spinal cord arranges in a certain way during disease processes and causes the patient pain [8]. There are input afferent sensory routes both from the musculoskeletal components and from the viscera themselves. The outputs are motor signals to the muscles and autonomic signals to the viscera, blood vessels, and glands present in that specific region [8]. Dysmenorrhea causes patients to present with spinal facilitation due to the impact that the menstrual cycle has on the spinal cord. Targeting the specific areas of the spinal cord impacted by dysmenorrhea could significantly improve these patients’ pain. 

Possible Case Scenario

The patient is a 35-year-old female who presents with dysmenorrhea secondary to endometriosis. History and physical examination reveal bilateral lower quadrant pain and tenderness, along with TART changes from T10 to L2. She is allergic to NSAIDs. She lives with her significant other, 50 miles away from the nearest clinic, and they do not own a vehicle. 

In this scenario, the patient is a good candidate for OMT, as an alternative to NSAIDs, OCPs, or an IUD. She lives in an area with poor healthcare access and has contraindications to traditional therapies. The physician could teach her significant other to apply sacral inhibition, which involves applying pressure to the sacral base for 90 seconds. This can be repeated for future episodes of dysmenorrheal pain without additional in-person clinic visits. This scenario highlights the benefits of OMT in treating patients with poor access to healthcare. When taught the appropriate treatments by physicians, family members can be involved in providing symptomatic relief. This decreases the burden of healthcare costs to the patient and provides an individualized treatment plan. 

## Conclusions

Numerous positive effects were found among these studies, including a reduction in the duration of pain, reduction of pain intensity, and reduction of analgesic use. However, the low number of studies supports the need for further investigations. Dysmenorrhea patients could benefit from future studies being performed with detailed methodology, descriptive summaries of techniques performed, and reporting of adverse events encountered to obtain reliable results that can be implemented by physicians treating future dysmenorrhea patients. These osteopathic philosophies can be used in dysmenorrhea patients to conduct a prospective RCT, targeting viscerosomatic reflexes and spinal facilitation to decrease pain duration, pain intensity, and analgesic use. Non-musculoskeletal (non-MSK)-focused OMT has a large body of mostly anecdotal evidence for relief of conditions such as migraine, GERD, and anxiety. It has helped when traditional standards of care have failed. Non-MSK-focused OMT research represents a relatively untouched field of research that can have a profound and positive global impact, particularly in areas with poor income/healthcare access. 
